# Author Correction: The bridge-like lipid transport protein VPS13C/PARK23 mediates ER–lysosome contacts following lysosome damage

**DOI:** 10.1038/s41556-026-02094-5

**Published:** 2026-09-23

**Authors:** Xinbo Wang, Peng Xu, Amanda Bentley-DeSousa, William Hancock-Cerutti, Shujun Cai, Benjamin T. Johnson, Francesca Tonelli, Lin Shao, Gabriel Talaia, Dario R. Alessi, Shawn M. Ferguson, Pietro De Camilli

**Affiliations:** 1 Department of Neuroscience, Yale University School of Medicine, New Haven, CT USA; 2 Department of Cell Biology, Yale University School of Medicine, New Haven, CT USA; 3 Howard Hughes Medical Institute, Yale University School of Medicine, New Haven, CT USA; 4 Program in Cellular Neuroscience, Neurodegeneration, and Repair, Yale University School of Medicine, New Haven, CT USA; 5 Aligning Science Across Parkinson’s (ASAP) Collaborative Research Network, Chevy Chase, MD USA; 6 MRC Protein Phosphorylation and Ubiquitylation Unit, School of Life Sciences, University of Dundee, Dundee, UK; 7 Center for Neurodevelopment and Plasticity, Wu Tsai Institute, Yale University, New Haven, CT USA

**Keywords:** Lysosomes, Mechanisms of disease

Correction to: *Nature Cell Biology* https://doi.org/10.1038/s41556-025-01653-6, published online 10 April 2025.

In the version of the article initially published, in Fig. 1d, one of the cells used to generate the histograms of this figure was accidentally counted 4 times. Thus, the total number of cells used to generate the figure is 57 and not 60, as previously indicated in the legend. This error had no noticeable impact on the histogram. The corresponding source data, the figure panel, and legend are now corrected.

In the statistical source data for Extended Data Fig. 4, which is meant to show the quantification of both LRRK1 cells and of TBK1/IKKe double KO cells, the quantification of the LRRK1 KO cells was accidentally shown twice. The correct file reporting the quantification of TBK1/IKKe cells is now provided. The figure panel was correct as published and was not changed. These corrections have been made to the HTML and PDF versions of the article.

