# Supplementary material for: The bridge-like lipid transport protein VPS13C/PARK23 mediates ER–lysosome contacts following lysosome damage
Source: Nat Cell Biol. 2025 Apr 10;27(5):776–89. doi: 10.1038/s41556-025-01653-6 (PMC12081312; doi:10.1038/s41556-025-01653-6)
Supplement: Supplementary file 1 — Supplementary Table 1 [file 41556_2025_1653_MOESM1_ESM.pdf]

nature cell biology

Supplementary information

https://doi.org/10.1038/s41556-025-01653-6

The bridge-like lipid transport protein VPS13C/PARK23 mediates ER–lysosome contacts following lysosome damage

In the format provided by the authors and unedited

Key Resource Table

RESOURCE TYPE	RESOURCE NAME	SOURCE	IDENTIFIER	NEW/REUSE	ADDITIONAL INFORMATION
Dataset	Source data	Zenodo	DOI: 10.5281/zenodo.14845889	new	Source data used in this research
Software	FIJI Version 2.14.0/1.54f	National Institute of Health (NIH)	https://imagej.net/software/fiji/; RRID: SCR_002285	reuse	
Software	GraphPad Prism 8	GraphPad Software, LLC	www.graphpad.com/; RRID: SCR_002798	reuse	
Software	StackReg	National Institute of Health (NIH)	https://bigwww.epfl.ch/thevenaz/stackreg/	reuse	
Software	Time Series Analyzer	National Institute of Health (NIH)	https://imagej.net/ij/plugins/time-series.html; (RRID:SCR_014269)	reuse	
Code	Script for image quantification	This work	DOI: 10.5281/zenodo.14814757	new	
Protocol	VPS13C^mClover-Flp-In cell line generation	protocols.io	DOI: dx.doi.org/10.17504/protocols.io.36wgqnnnxgk5/v1	new	
Antibody	VPS13C	Proteintech	Cat# 29844-1-AP; RRID: AB_3086177	new	Dilution: 1:1000
Antibody	Rab7	Cell Signaling Technology	Cat# 9367; RRID: AB_1904103	new	Dilution: 1:1000
Antibody	Mouse monoclonal anti-Rab7A	Sigma Aldrich	Cat# R8779; RRID:AB_609910	new	Dilution: 1:2000
Antibody	pRab7 (S72)	Abcam	Cat# ab302494; RRID: AB_2933985	new	Dilution: 1:1000
Antibody	TBK1	Cell Signaling Technologies	Cat# 3504S; RRID: AB_2255663	new	Dilution: 1:2000
Antibody	pTBK1(S172)	Cell Signaling Technologies	Cat# 5483S; RRID: AB_10693472	new	Dilution: 1:1000
Antibody	Lamp1	Cell Signaling Technologies	Cat# 9091; RRID: AB_2687579	new	Dilution: 1:2000
Antibody	Lamp1	Abcam	Cat# ab25630; RRID:AB_470708	new	Dilution: 1:100
Antibody	Galectin3	R&D Systems	Cat# IC1154G; RRID:AB_10890949	new	Dilution: 1:50
Antibody	GM130	BD Biosciences	Cat# 610822; RRID: AB_398141	new	Dilution: 1:2000
Antibody	PDI	Cell Signaling Technologies	Cat# 2446S; RRID: AB_2298935	new	Dilution: 1:1000
Antibody	VAPB	Sigma Aldrich	Cat# HPA013144; RRID: AB_1858717	new	Dilution: 1:4000
Antibody	mCherry	Abcam	Cat# Ab125096; RRID: AB_11133266	new	Dilution: 1:1000
Antibody	Tubulin	Sigma Aldrich	Cat# T5168; RRID: AB_477579	new	Dilution: 1:2000
Antibody	GFP	Abcam	Cat# ab290; RRID: AB_303395	new	Dilution: 1:1000
Antibody	GAPDH	Proteus	Cat# 40-1246	new	Dilution: 1:1000
Antibody	LRRK1	MRC Reagents and Services	Cat# S405C	new	Final concentration: 1 µg/mL
Antibody	IKKe	Cell Signaling Technology	Cat# 3416S; RRID:AB_1264180	new	Dilution: 1:2000
Chemical	LLOMe	Sigma Aldrich	Cat# L7393-500MG; CAS: 16689-14-8	new	
Chemical	Chloroquine	Sigma Aldrich	Cat# C6628, CAS: 50-63-5	new	
Chemical	Nigericin	Sigma Aldrich	Cat# N7143, CAS: 28643-80-3	new	
Chemical	Saliphenylhalamide	Omm Scientific	N/A	new	
Chemical	OSW-1	MedChem Express	Cat# HY-101213, CAS: 145075-81-6	new	
Chemical	LysoView640	Biotium	Cat# 70058	new	
Chemical	LysoView633	Biotium	Cat# 70085	new	
Chemical	Tetracycline	Thermo Scientific	Cat# A39246, CAS: 64-75-5	new	
Chemical	E64d	Cayman Chemical	Cat# 13533, CAS: 88321-09-9	new	
Chemical	Lipofectamine RNAiMAX	Thermo Scientific	Cat# 13778030	new	
Chemical	IRdye 800CW	LI-COR Biosciences	Cat# 926-32213, RRID:AB_621848	new	
Chemical	IRdye 680LT	LI-COR Biosciences	Cat# 926-68020, RRID:AB_10706161	new	
Chemical	FuGene HD	Promega	Cat# E2311	new	
Oligonucleotide	Human RAB7A siRNA	Horizon Biosciences	Cat# M-010388-00-0005	new	Final concentration:100nM
Cell line	A549	ATCC	RRID:CVCL_0023		
Cell line	RPE1	ATCC	Cat# CRL-4000; RRID:CVCL_4388		
Cell line	HeLa	Cellosaurus	Cat# RCB5388; RRID:CVCL_R965		
Cell line	WT mouse embryonic fibroblasts	dx.doi.org/10.17504/protocols.io.eq2ly713qlx9/v1			
Cell line	LRRK1 KO mouse embryonic fibroblasts	dx.doi.org/10.17504/protocols.io.eq2ly713qlx9/v1			
Cell line	VPS13C^mClover-Flp-In	This work	RRID:CVCL_E6IS		
Cell line	A549 VPS13C KO	This work	RRID:CVCL_E6IP		
Cell line	Hela VPS13C KO	https://doi.org/10.1083/jcb.202106046	RRID:CVCL_E6IQ		
Cell line	Hela Rab7 KO	https://doi.org/10.1038/s44318-024-00180-8	RRID:CVCL_D7ET		
plasmid	pmScarlet-PPM1H	Addgene	RRID:Addgene_233585		
plasmid	pmScarlet-LRRK1 K746G	Addgene	RRID:Addgene_233586		
plasmid	VPS13C^Halo	Addgene	RRID:Addgene_232864		
plasmid	Halo-SspB-PPM1H	Addgene	RRID:Addgene_232870		
plasmid	VPS13C^Halo-Δ(ATG2C-PH)	Addgene	RRID:Addgene_232867		
plasmid	mCherry-ATG2C(VPS13C)	Addgene	RRID:Addgene_232868		
plasmid	mCherry-PH(VPS13C)	Addgene	RRID:Addgene_232869		
plasmid	mCherry-VAB(VPS13C)	Addgene	RRID:Addgene_232865		
plasmid	mCherry-VPS13C-Cter	Addgene	RRID:Addgene_232866		
plasmid	mCherry-OSBP	Addgene	RRID:Addgene_232871		
plasmid	GFP-LRRK2	Addgene	RRID:Addgene_232872		
plasmid	3xflag-CMV10	Sigma Aldrich	Cat# E7658		
plasmid	HA-PPM1H	MRC Reagents and Services	DU62789		
plasmid	VPS13C^mClover3	Addgene	RRID:Addgene_118760		
plasmid	mCherry-VAPB	Addgene	RRID:Addgene_108126		
plasmid	GFP-LRRK1	MRC Reagents and Services	DU30382		
plasmid	GFP-LRRK1 K746G	MRC Reagents and Services	DU67083		
plasmid	GFP-LRRK1 D1409A	MRC Reagents and Services	DU67084		
plasmid	pmScarlet	Addgene	RRID:Addgene_85044		
plasmid	mCherry-SopF	Addgene	RRID:Addgene_135174		
plasmid	LAMP1-mCherry-iLID	Addgene	RRID:Addgene_174625		
plasmid	Lamp1-RFP	Addgene	RRID:Addgene_1817		
plasmid	mCherry-Gal3	Addgene	RRID:Addgene_85662		
plasmid	Flp-In TREx 293	Invitrogen	RRID:CVCL_U427		
plasmid	pcDNA5/FRT/TO	Addgene	RRID:Addgene_41000		
plasmid	pOG44	Addgene	RRID:Addgene_209087		
plasmid	pcDNA5/FRT	Invitrogen	Cat# V601020		
plasmid	IST1-Apple	a gift from Phyllis Hanson, University of Michigan School of Medicine, Ann Arbor, MI.			
