# Supplementary material for: The bridge-like lipid transport protein VPS13C/PARK23 mediates ER–lysosome contacts following lysosome damage
Source: Nat Cell Biol. 2025 Apr 10;27(5):776–89. doi: 10.1038/s41556-025-01653-6 (PMC12081312; doi:10.1038/s41556-025-01653-6)
Supplement: Supplementary file 2 — Reporting Summary [file 41556_2025_1653_MOESM2_ESM.pdf]

nature portfolio

Corresponding author(s): Pietro De Camilli
Last updated by author(s): Feb 11, 2025

Reporting Summary

Nature Portfolio wishes to improve the reproducibility of the work that we publish. This form provides structure for consistency and transparency in reporting. For further information on Nature Portfolio policies, see our Editorial Policies and the Editorial Policy Checklist.

Statistics

For all statistical analyses, confirm that the following items are present in the figure legend, table legend, main text, or Methods section.

n/a	Confirmed	
☐	☒	The exact sample size (*n*) for each experimental group/condition, given as a discrete number and unit of measurement
☐	☒	A statement on whether measurements were taken from distinct samples or whether the same sample was measured repeatedly
☐	☒	The statistical test(s) used AND whether they are one- or two-sided<br>*Only common tests should be described solely by name; describe more complex techniques in the Methods section.*
☒	☐	A description of all covariates tested
☒	☐	A description of any assumptions or corrections, such as tests of normality and adjustment for multiple comparisons
☐	☒	A full description of the statistical parameters including central tendency (e.g. means) or other basic estimates (e.g. regression coefficient) AND variation (e.g. standard deviation) or associated estimates of uncertainty (e.g. confidence intervals)
☐	☒	For null hypothesis testing, the test statistic (e.g. *F*, *t*, *r*) with confidence intervals, effect sizes, degrees of freedom and *P* value noted<br>*Give P values as exact values whenever suitable.*
☒	☐	For Bayesian analysis, information on the choice of priors and Markov chain Monte Carlo settings
☒	☐	For hierarchical and complex designs, identification of the appropriate level for tests and full reporting of outcomes
☒	☐	Estimates of effect sizes (e.g. Cohen's *d*, Pearson's *r*), indicating how they were calculated

Our web collection on statistics for biologists contains articles on many of the points above.

Software and code

Policy information about availability of computer code

Data collection	Western blots were imaged on the Odyssey imaging system (LI-COR, ODY-2461); Fluorescence images were captured using a Nikon microscope (Yokogawa CSU-W1 SoRa, Nikon)
Data analysis	All immunoblot data and fluorescence images were processed and analyzed with ImageJ/Fiji (Version 2.14.0/1.54f). Statistical analysis was carried out with GraphPad Prism version 8.0.1

For manuscripts utilizing custom algorithms or software that are central to the research but not yet described in published literature, software must be made available to editors and reviewers. We strongly encourage code deposition in a community repository (e.g. GitHub). See the Nature Portfolio guidelines for submitting code & software for further information.

Data

Policy information about availability of data

All manuscripts must include a data availability statement. This statement should provide the following information, where applicable:

- Accession codes, unique identifiers, or web links for publicly available datasets
- A description of any restrictions on data availability
- For clinical datasets or third party data, please ensure that the statement adheres to our policy

Data is available on Zenodo, DOI:10.5281/zenodo.14846056

Research involving human participants, their data, or biological material

Policy information about studies with human participants or human data. See also policy information about sex, gender (identity/presentation), and sexual orientation and race, ethnicity and racism.

Reporting on sex and gender	n/a
Reporting on race, ethnicity, or other socially relevant groupings	n/a
Population characteristics	n/a
Recruitment	n/a
Ethics oversight	n/a

Note that full information on the approval of the study protocol must also be provided in the manuscript.

Field-specific reporting

Please select the one below that is the best fit for your research. If you are not sure, read the appropriate sections before making your selection.

☒ Life sciences ☐ Behavioural & social sciences ☐ Ecological, evolutionary & environmental sciences

For a reference copy of the document with all sections, see nature.com/documents/nr-reporting-summary-flat.pdf

Life sciences study design

All studies must disclose on these points even when the disclosure is negative.

Sample size	No statistical methods were used to predetermine sample sizes but our sample sizes are similar to those reported in previous studies in this field. The sample size and replications for each experiment is specified in the figure legends.
Data exclusions	No data were excluded.
Replication	All experiments were performed at least in triplicate unless indicated otherwise in the figure legends.
Randomization	For fluorescence microscopy image acquisition, cells were selected at random microscopy fields.
Blinding	Data analysis was not performed blind to the experimental conditions. Most cell-based quantifications were conducted using live-cell imaging from time-lapse movies, ensuring internal controls. Additionally, the quantification methods relied on software-based automatic calculations.

Reporting for specific materials, systems and methods

We require information from authors about some types of materials, experimental systems and methods used in many studies. Here, indicate whether each material, system or method listed is relevant to your study. If you are not sure if a list item applies to your research, read the appropriate section before selecting a response.

Materials & experimental systems

n/a	Involved in the study	
☐	☒	Antibodies
☐	☒	Eukaryotic cell lines
☒	☐	Palaeontology and archaeology
☐	☒	Animals and other organisms
☒	☐	Clinical data
☒	☐	Dual use research of concern
☒	☐	Plants

Methods

n/a	Involved in the study	
☒	☐	ChIP-seq
☒	☐	Flow cytometry
☒	☐	MRI-based neuroimaging

Antibodies

Antibodies used	Anti-LAMP1 (Cell Signaling Technology, 9091; RRID: AB_2687579, for WB, 1:2000)<br>anti-LAMP1 (Abcam, ab25630; RRID:AB_470708, for IF, 1:100)<br>anti-Galectin3 (R&D Systems, IC1154G; RRID:AB_10890949, for IF, 1:50)

anti-VPS13C (Proteintech, 29844-1-AP; RRID: AB_3086177, for WB, 1:1000)
anti-GM130 (BD Biosciences, 610822; RRID: AB_398141, for WB, 1:2000)
anti-PDI (CST, 2446S; RRID: AB_2298935, for WB, 1:1000)
anti-VAPB (Sigma-Aldrich, HPA013144; RRID: AB_1858717, for WB 1:4000)
anti-Rab7 (Cell Signaling Technology, 9367; RRID: AB_1904103, for WB, 1:1000)
anti-Rab7A (Sigma Aldrich, R8779; RRID:AB_609910, for WB: 1:2000)
anti-pSer72 Rab7(Abcam, ab302494; RRID: AB_2933985, for WB, 1:1000)
anti-GAPDH (Proteus, 40-1246; for WB, 1:1000)
anti-GFP (Abcam, ab290; RRID: AB_303395, for WB, 1:1000)
anti-tubulin (Sigma Aldrich, T5168; RRID: AB_477579, for WB, 1:2000)
anti-mCherry (Abcam, Ab125096; RRID: AB_11133266, for WB, 1:1000)
anti-LRRK1 (MRC Reagents and Services, S405C; for WB: 1 µg/mL)
anti-IKKe (Cell Signaling Technology, 3416S; for WB: 1:2000)
anti-TBK1 (Cell Signaling Technology, 3504S; RRID: AB_2255663, for WB, 1:2000)
anti-pSer172 TBK1 (Cell Signaling Technology, 5483S; RRID: AB_10693472, for WB, 1:1000)

Validation

All antibodies used in this study were commercially obtained and validated either by the suppliers, previous studies, or this study. Validation was based on factors such as the molecular weight of detected bands, the use of samples from knockout or knockdown cells or mice, and the reproducibility of results.

Anti-LAMP1 (Cell Signaling Technology, 9091; RRID: AB_2687579)-PMID:37487100; Vendor:https://www.cellsignal.com/products/primary-antibodies/lamp1-d2d11-xp-rabbit-mab/9091?srsltid=AfmBOor2rf0T6V7V_SF7MJ-VwEkEmKGP5wdIHSi6b1ZSEYrBmrn1O9nr
anti-LAMP1 (Abcam, ab25630; RRID:AB_470708)-PMID: 38020041; Vendor:https://www.abcam.com/en-us/products/primary-antibodies/lamp1-antibody-h4a3-ab25630?srsltid=AfmBOopuGERLMpIH-CAFS6UpjtwGVuCg01tX0sKyRfivIqkowmgua_bb
anti-Galectin3 (R&D Systems, IC1154G; RRID:AB_10890949)-PMID: 30314966; Vendor:https://www.rndsystems.com/products/human-galectin-3-alexa-fluor-488-conjugated-antibody_ic1154g
anti-VPS13C (Proteintech, 29844-1-AP; RRID: AB_3086177)-PMID: 35657605; Vendor:https://www.ptglab.com/products/VPS13C-Antibody-29844-1-AP.htm?srsltid=AfmBOoo7mg91LAtgih8Uf9MdJSgPwYX4F62oWOBTRcHm-EXmgkLlCw59
anti-GM130 (BD Biosciences, 610822; RRID: AB_398141)-PMID: 35650196; Vendor:https://www.bdbiosciences.com/en-us/products/reagents/microscopy-imaging-reagents/immunofluorescence-reagents/purified-mouse-anti-gm130.610822?tab=product_details
anti-PDI (CST, 2446S; RRID: AB_2298935)-PMID: 39080411; Vendor:https://www.cellsignal.com/products/primary-antibodies/pdi-antibody/2446?srsltid=AfmBOorZt6OJ-7cgnWmLQuih3acQCQt14XDH9qdW6SwTxUlVT6wy1QZE
anti-VAPB (Sigma-Aldrich, HPA013144; RRID: AB_1858717)-PMID:37528084; Vendor:https://www.sigmaaldrich.com/US/en/product/sigma/hpa013144?srsltid=AfmBOor3xkJB5wqu0yyUwFUniVVjhTm_x89uOG7qM5W1rwK-iMQAm2ft
anti-Rab7 (Cell Signaling Technology, 9367; RRID: AB_1904103)-PMID: 37141099; Vendor:https://www.cellsignal.com/products/primary-antibodies/rab7-d95f2-xp-rabbit-mab/9367?srsltid=AfmBOooOm-51XTvpHu3vtk-u9Er9N1M5JoWIC3kmCYL9IhfeUCG7qMCv
anti-Rab7A (Sigma Aldrich, R8779; RRID:AB_609910)-PMID: 24145164; Vendor:https://www.sigmaaldrich.com/US/en/product/sigma/r8779?srsltid=AfmBOorcquFbixad2UwJ6cX_Z4fZzGPL4K7hAFT1LJOBV6Wmt1o6ztEM
anti-pSer72 Rab7(Abcam, ab302494; RRID: AB_2933985)-PMID: 37141099; Vendor:https://www.abcam.com/en-us/products/primary-antibodies/rab7-phospho-s72-antibody-mjf-r38-1-ab302494
srsltid=AfmBOop8aqeHGAmnyDRlHDvu_JxVABZ5WHWknoLOl3r7DNBj6U-Px5Fv
anti-GAPDH (Proteus, 40-1246)-PMID: 39331042;
anti-GFP (Abcam, ab290; RRID: AB_303395); Vendor:https://www.abcam.com/en-us/products/primary-antibodies/gfp-antibody-ab290?srsltid=AfmBOoofiEkHQYrBdlGtQYifQBBASyBj6N5Ev8YQZVA2iy4cHxXfze5O
anti-tubulin (Sigma Aldrich, T5168; RRID: AB_477579)-PMID: 39386594; Vendor:https://www.sigmaaldrich.com/US/en/search/t5168?focus=products&page=1&perpage=30&sort=relevance&term=t5168&type=product
anti-mCherry (Abcam, Ab125096; RRID: AB_11133266)-PMID: 27447450; Vendor:https://www.abcam.com/en-us/products/primary-antibodies/mcherry-antibody-1c51-ab125096?srsltid=AfmBOop5kY_4E_vgDxPlMXAifkvtNuo9AW6Kz2BFusNV4_75j7XDPjEv
anti-LRRK1 (MRC Reagents and Services, S405C)-PMID: 33459343; Vendor:https://mrcppureagents.dundee.ac.uk/reagents-view-antibodies/588116
anti-IKKe (Cell Signaling Technology, 3416S)-PMID: 37595039; Vendor:https://www.cellsignal.com/products/primary-antibodies/ikke-d61f9-xp-rabbit-mab/3416?srsltid=AfmBOoqB2t9sqU2w8PD0ufk0O46318GWXTUuXk--5xzFUe796J73TDvx
anti-TBK1 (Cell Signaling Technology, 3504S; RRID: AB_2255663)-PMID: 37595039; Vendor:https://www.cellsignal.com/products/primary-antibodies/tbk1-nak-d1b4-rabbit-mab/3504?srsltid=AfmBOoo2vLldCH6jHEY4x-xr-4h6E_6fKnKlr2Dw9fz5qU55A4pIf3fT
anti-pSer172 TBK1 (Cell Signaling Technology, 5483S; RRID: AB_10693472)-PMID: 39712456; Vendor:https://www.cellsignal.com/products/primary-antibodies/phospho-tbk1-nak-ser172-d52c2-xp-rabbit-mab/5483?srsltid=AfmBOop2-rUaa7Jn0erfxFOZtgWbifhqglJIsb47EBUazZUraG5qSNPE

Eukaryotic cell lines

Policy information about cell lines and Sex and Gender in Research

Cell line source(s)

RPE1 cells (ATCC, RRID:CVCL_4388); HeLa (RRID:CVCL_R965); Flp-In TREx 293 (Invitrogen); A549 cells (ATCC, RRID:CVCL_0023)

Authentication

Cell lines obtained were not authenticated

Mycoplasma contamination

Cells were tested negative for mycoplasma contamination using PCR-based assays (Lonza Mycoplasma kit) .

Commonly misidentified lines
(See ICLAC register)

No commonly misidentified cell lines was used.

Animals and other research organisms

Policy information about studies involving animals; ARRIVE guidelines recommended for reporting animal research, and Sex and Gender in Research

Laboratory animals	Mice were maintained under specific pathogen-free conditions and housed at an ambient temperature (20–24°C) and humidity (45–55%) and maintained on a 12 h light/12 h dark cycle, with free access to food (SDS RM No. 3 autoclavable) and water.
Wild animals	No wild animals were used in this study
Reporting on sex	The gender was not considered in this study
Field-collected samples	No filed collected samples in this study
Ethics oversight	Mice studies were ethically reviewed and carried out in accordance with Animals (Scientific Procedures) Act 1986 and regulations set by the University of Dundee and the U.K. Home Office. All mice studies and breeding were approved by the University of Dundee ethical committee and performed under a U.K. Home Office project license.

Note that full information on the approval of the study protocol must also be provided in the manuscript.

Plants

Seed stocks	n/a
Novel plant genotypes	n/a
Authentication	n/a
