# Supplementary figures and images for: The bridge-like lipid transport protein VPS13C/PARK23 mediates ER–lysosome contacts following lysosome damage
Source: Nat Cell Biol. 2025 Apr 10;27(5):776–89. doi: 10.1038/s41556-025-01653-6 (PMC12081312; doi:10.1038/s41556-025-01653-6)

Source data Figure 1

Fig. 1F

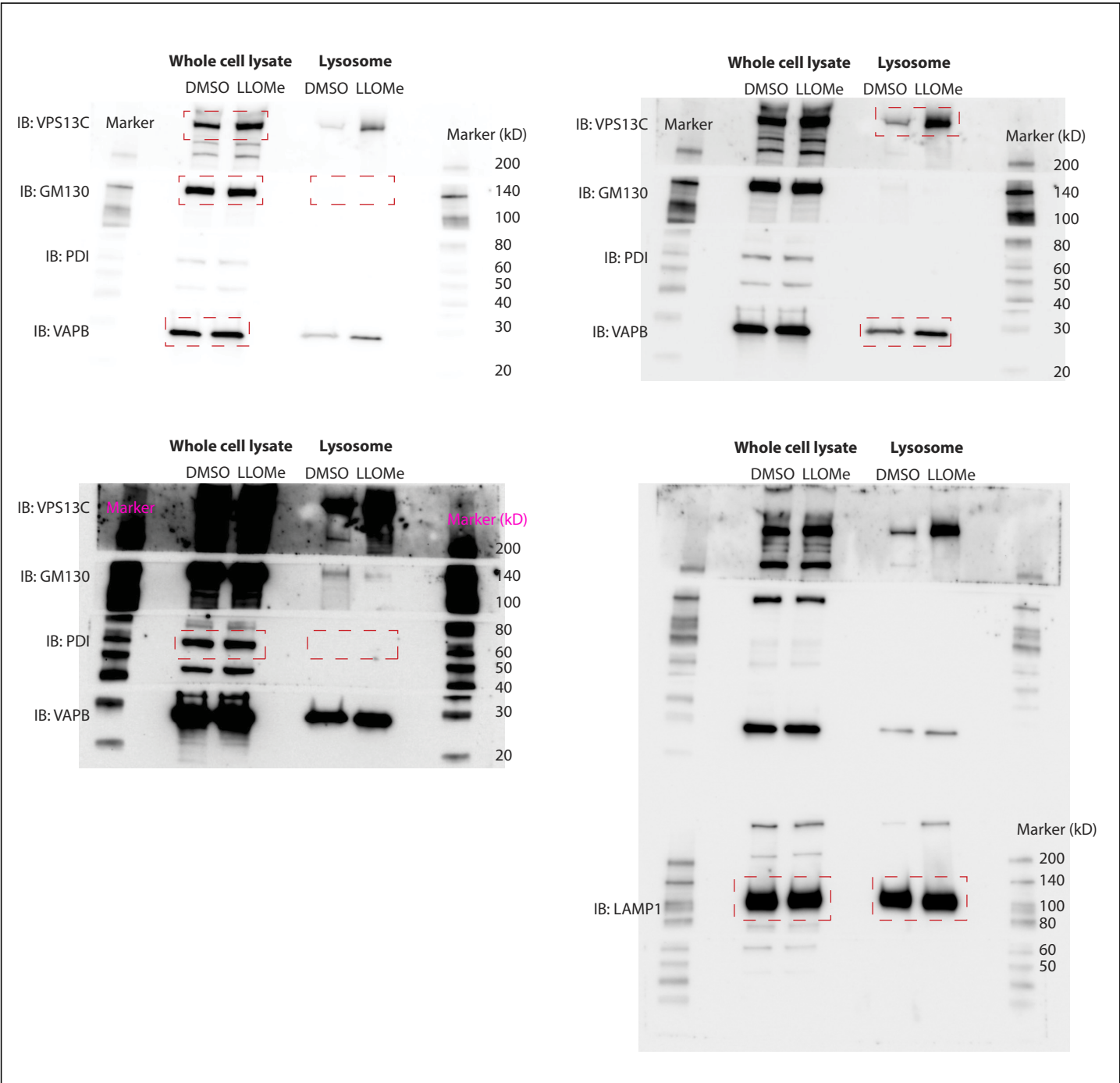

Supplement: Supplementary file 5 — Unprocessed western blots. [file 41556_2025_1653_MOESM5_ESM.pdf]

Source data Figure 3

Fig. 3A      Fig. 3D

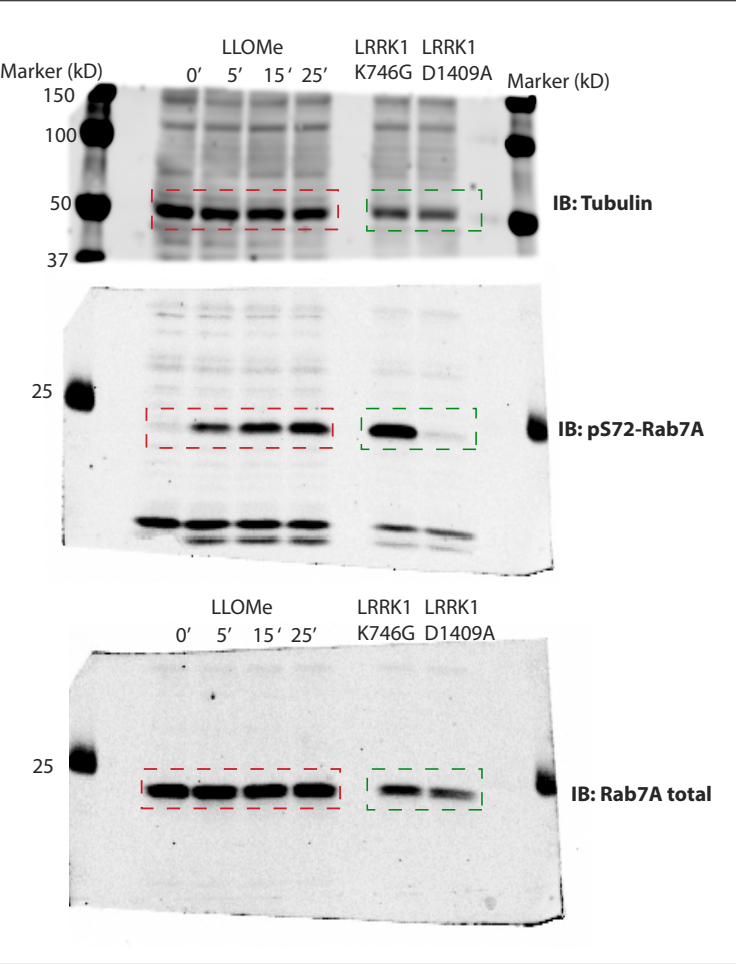

Fig. 3B

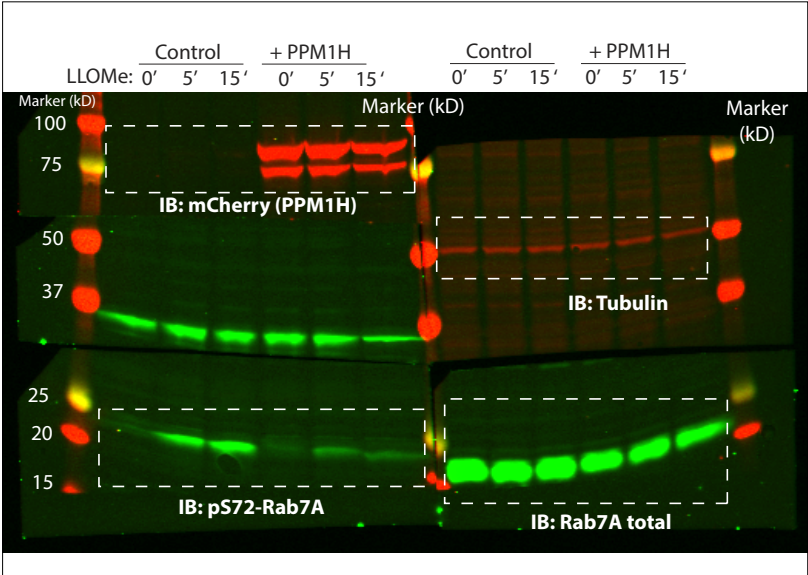

Supplement: Supplementary file 8 — Unprocessed western blots. [file 41556_2025_1653_MOESM8_ESM.pdf]

### Extended Data Fig. 1B

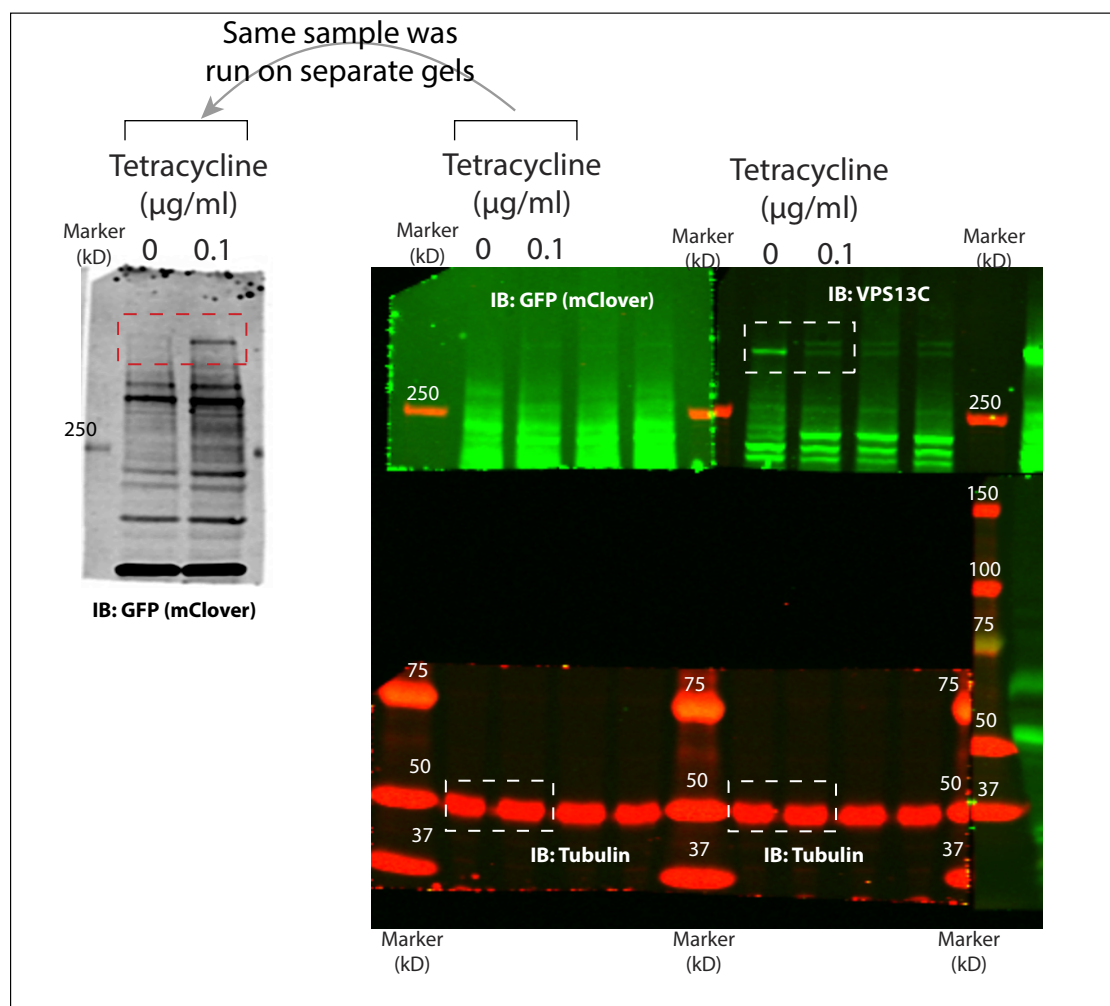

Supplement: Supplementary file 13 — Unprocessed western blots. [file 41556_2025_1653_MOESM13_ESM.pdf]

Extended Data Fig. 3A

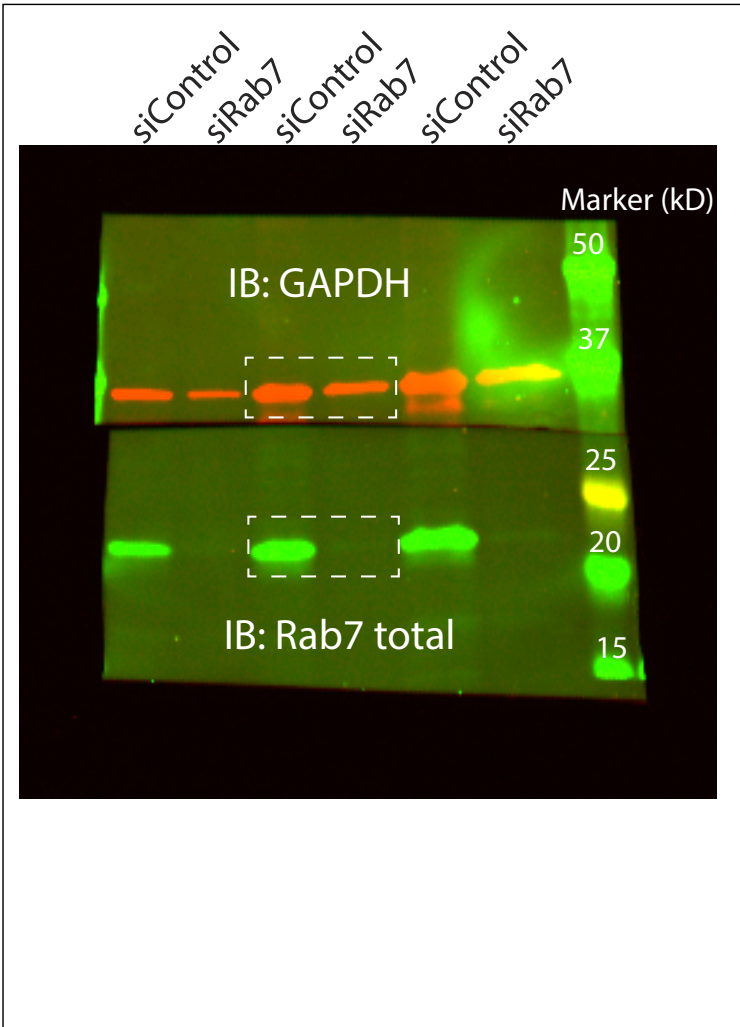

Extended Data Fig. 3B

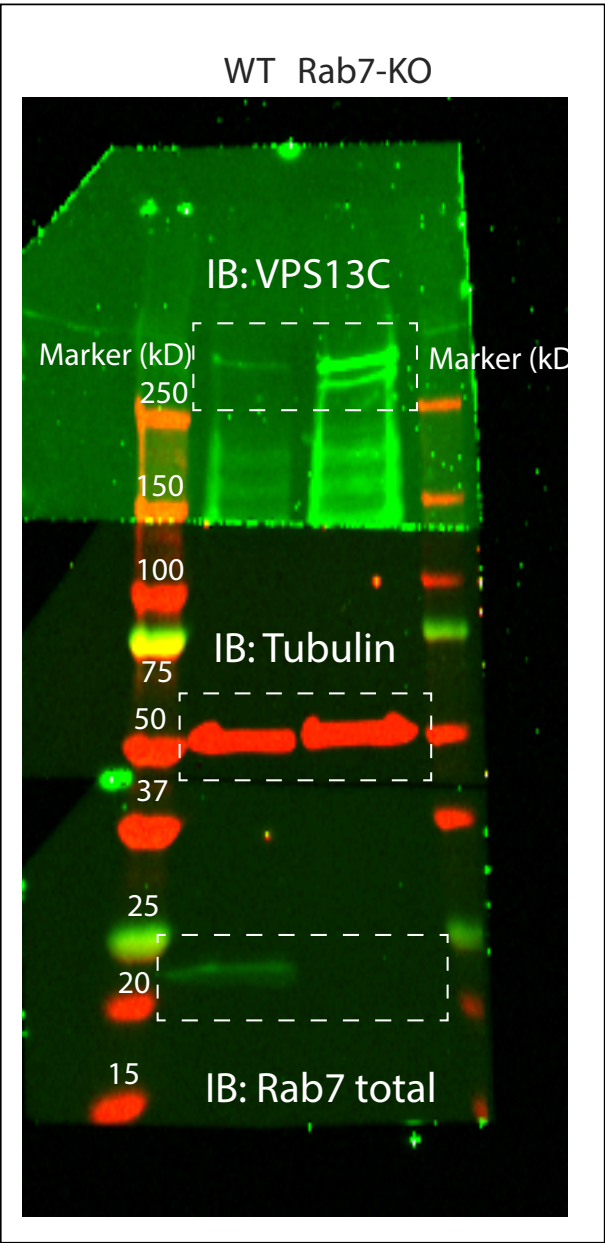

Supplement: Supplementary file 15 — Unprocessed western blots. [file 41556_2025_1653_MOESM15_ESM.pdf]

Source data Extended Data Figure 4

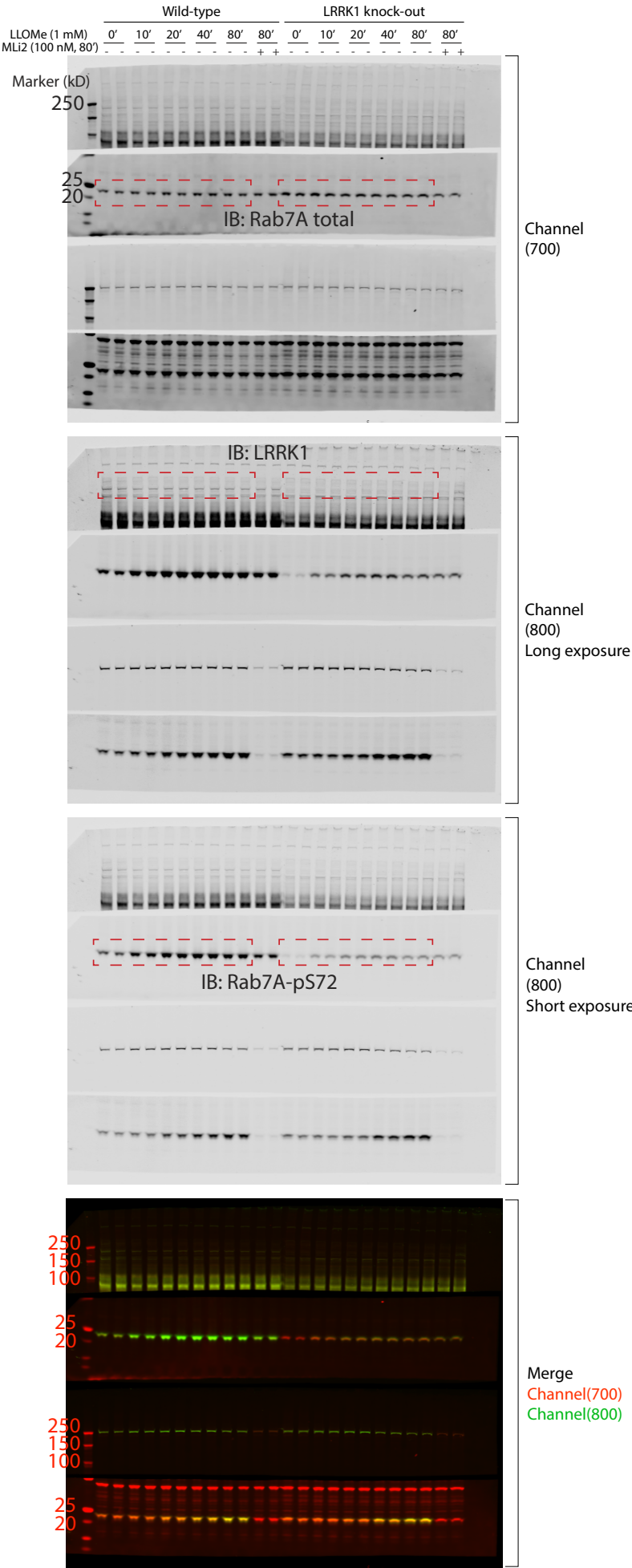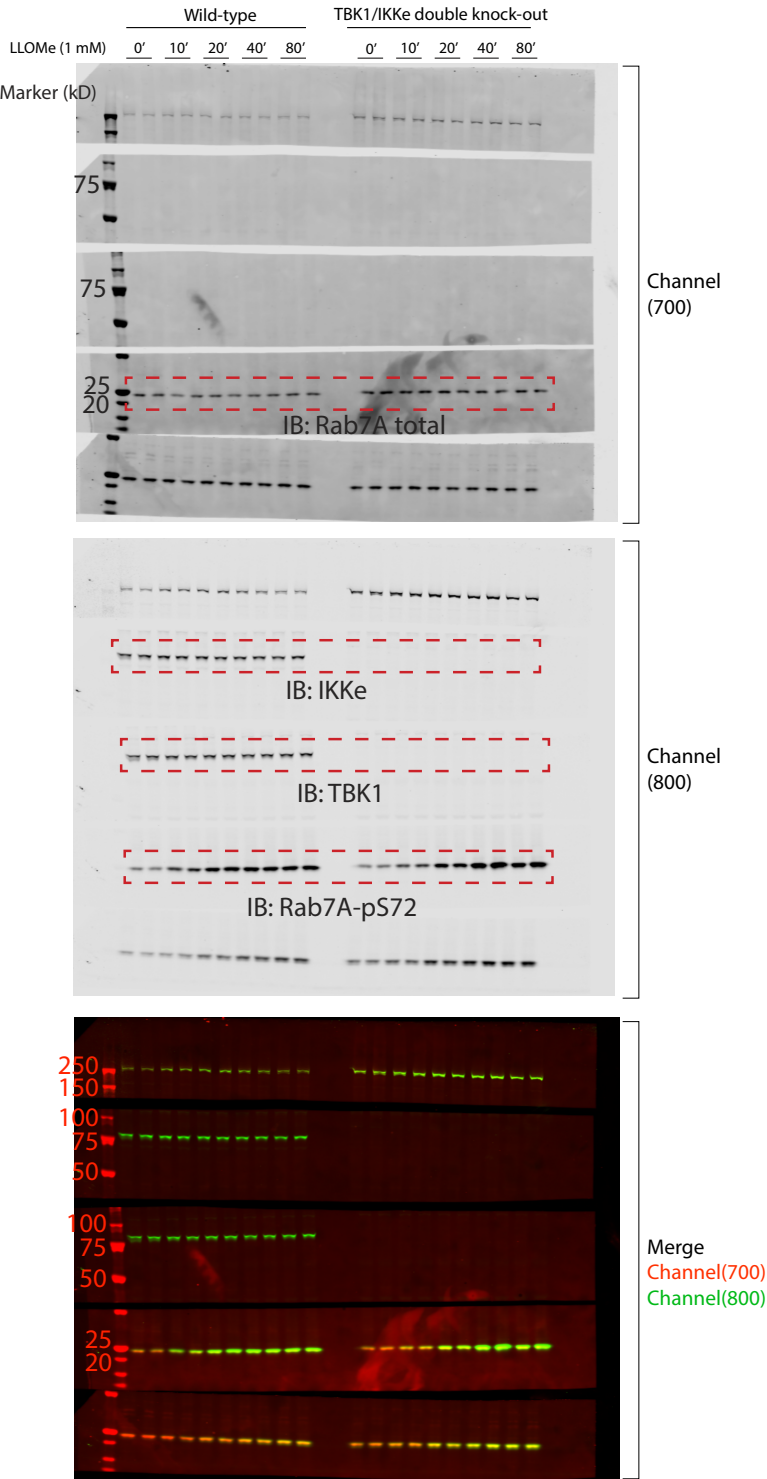

Supplement: Supplementary file 17 — Unprocessed western blots. [file 41556_2025_1653_MOESM17_ESM.pdf]

Source data Extended Data Figure 8

Extended Data Fig. 8A

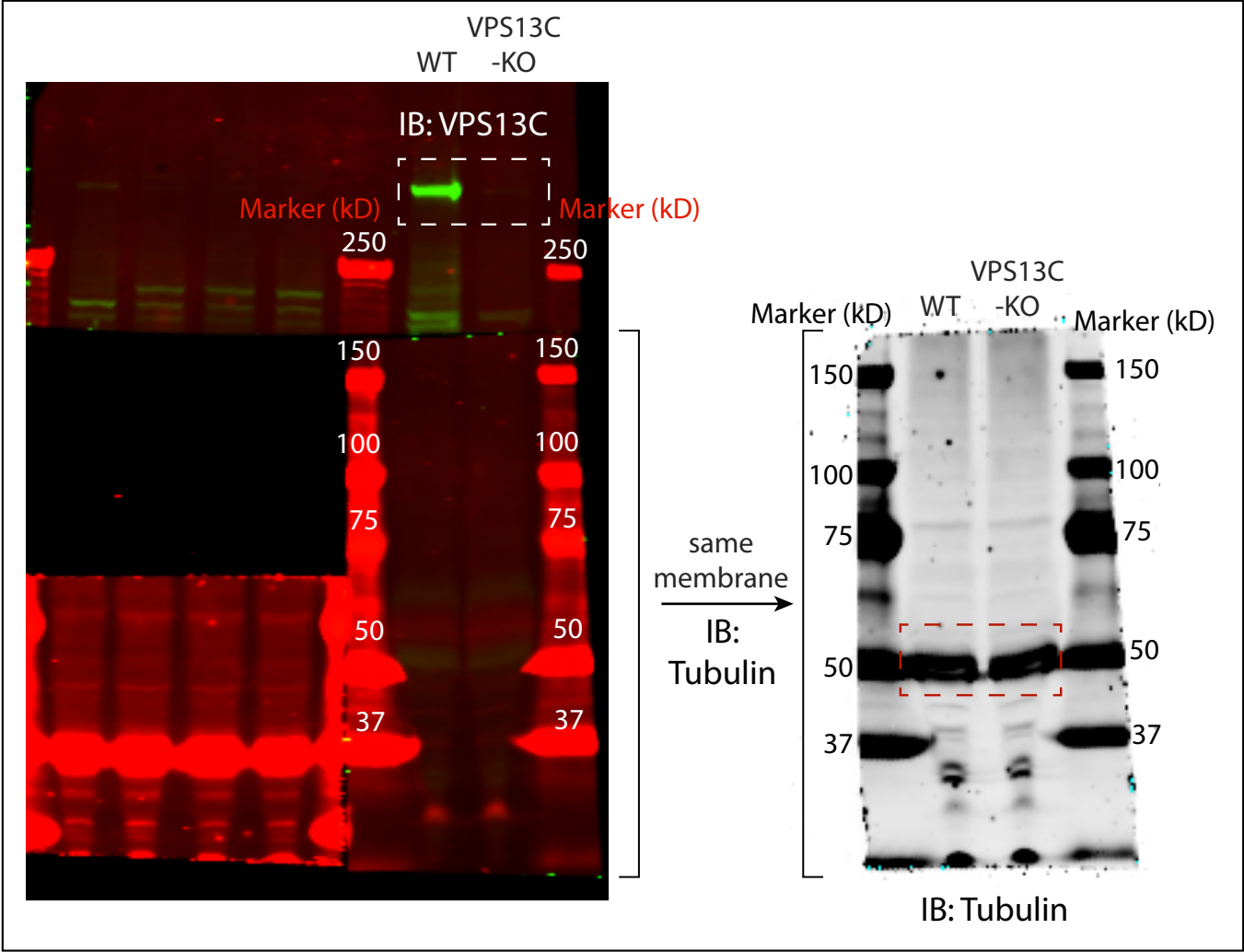

Supplement: Supplementary file 18 — Unprocessed western blots. [file 41556_2025_1653_MOESM18_ESM.pdf]
